# Acute Upper Airway Obstruction Induced by Nasogastric Tube Syndrome During the Management of a Bowel Obstruction

**DOI:** 10.7759/cureus.106787

**Published:** 2026-04-10

**Authors:** Senthil Rajamanickam, Anurag Agarwal, Eid-Christian Oweis, Suhaib Ahmad

**Affiliations:** 1 Department of Otolaryngology, Head and Neck Surgery, Betsi Cadwaladr University Health Board, Bangor, GBR; 2 General Surgery, Betsi Cadwaladr University Health Board, Bangor, GBR; 3 Department of Orthopaedics, Betsi Cadwaladr University Health Board, Bangor, GBR; 4 Department of General Surgery, Health Education and Improvement Wales (HEIW), Bangor, GBR

**Keywords:** acute airway obstruction, bariatric surgery and small bowel obstruction, difficult airway management, ng tube, ryles tube

## Abstract

A man in his sixties developed acute upper airway obstruction five days after nasogastric tube insertion for conservative management of small bowel obstruction. Initial symptoms were subtle, with preserved oxygen saturation and phonation despite progressive airway compromise. Flexible nasal endoscopy demonstrated marked supraglottic and hypopharyngeal oedema, and contrast-enhanced CT confirmed severe supraglottic narrowing without evidence of abscess or mass lesion. Despite prompt treatment with systemic corticosteroids and nebulised adrenaline, respiratory distress progressed, necessitating awake fibreoptic intubation, intensive care admission, and subsequent surgical tracheostomy. Gradual clinical improvement followed, with resolution of airway oedema and recovery of voice and swallowing function prior to discharge. This case highlights nasogastric tube syndrome as an under-recognised cause of delayed, life-threatening airway obstruction.

## Introduction

Nasogastric tube insertion is a routine intervention in the management of gastrointestinal obstruction, postoperative ileus, and nutritional support. Although generally considered safe, it is associated with a range of complications, most of which are minor and self-limiting. Rarely, however, nasogastric tube placement may precipitate severe upper airway compromise, a condition referred to as nasogastric tube syndrome [1].

Nasogastric tube syndrome is characterised by supraglottic and hypopharyngeal oedema, vocal cord dysfunction, and progressive airway obstruction occurring during or after nasogastric intubation. Since its initial description, the condition has been reported infrequently in the literature, suggesting it is under-recognised rather than truly rare [2]. Early symptoms are often subtle and may include throat discomfort, hoarseness, or mild dyspnoea, frequently in the absence of hypoxia. As a result, the diagnosis may be delayed or misattributed to lower respiratory tract infection, allergic reaction, or bronchospasm [3].

Delayed recognition is clinically significant, as nasogastric tube syndrome may progress rapidly to life-threatening airway obstruction. Mortality has been reported, particularly when escalation of airway management is delayed [4]. Awareness of this entity is therefore essential, especially among surgical and acute care clinicians, to facilitate prompt investigation, early otolaryngology involvement, and timely airway protection. This case highlights the diagnostic challenge posed by nasogastric tube syndrome and reinforces the importance of maintaining a high index of suspicion in patients who develop respiratory symptoms following nasogastric tube insertion [5].

## Case presentation

A man in his sixties, with a history of ischaemic heart disease, hypertension, gastro-oesophageal reflux disease and previous emergency appendicectomy, presented with abdominal pain, vomiting and absence of stoma output for 24 hours. He had a history of mid-rectal adenocarcinoma treated with total neoadjuvant chemoradiotherapy followed by local radiotherapy, with subsequent local recurrence managed surgically by abdominoperineal resection and end colostomy. His postoperative recovery had initially been uncomplicated.

Several weeks later, he re-presented with features of small bowel obstruction. CT demonstrated dilated small bowel loops with a transition point near the terminal ileum. Conservative management was commenced, including intravenous fluid resuscitation and nasogastric decompression. A 16-French nasogastric tube was inserted via the right nostril. Two initial attempts were unsuccessful due to resistance at approximately 8 cm. A third attempt was successful and advanced to the predetermined depth. There was no bleeding, coughing or coiling noted during insertion, and minimal gastric aspirate was obtained.

Over the following days, abdominal symptoms improved. Nasogastric output remained low, and a contrast challenge confirmed resolution of the obstruction with return of stoma output. During this period, the patient developed new respiratory symptoms, including chest discomfort, hiccups and frothy sputum production. Observations remained stable on room air, and he was able to speak in full sentences without dysphagia.

**Figure 1:**
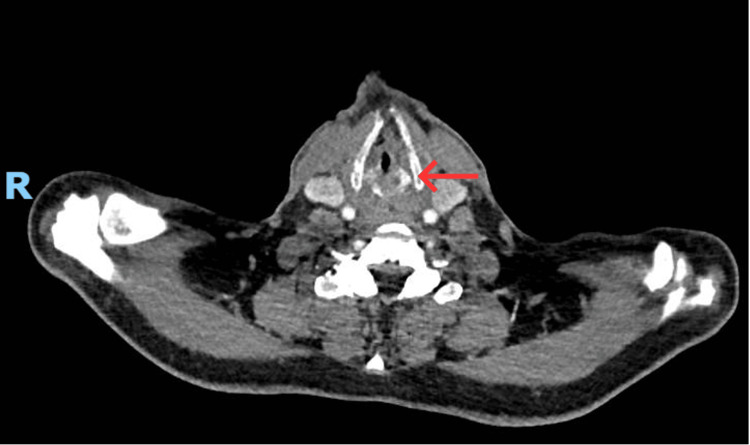
Axial CT of the neck at the level of the thyroid cartilage obtained during stridor, demonstrating bilateral vocal cord, laryngeal and hypopharyngeal oedema. The red arrow highlights circumferential soft-tissue swelling causing marked airway narrowing.

**Figure 2:**
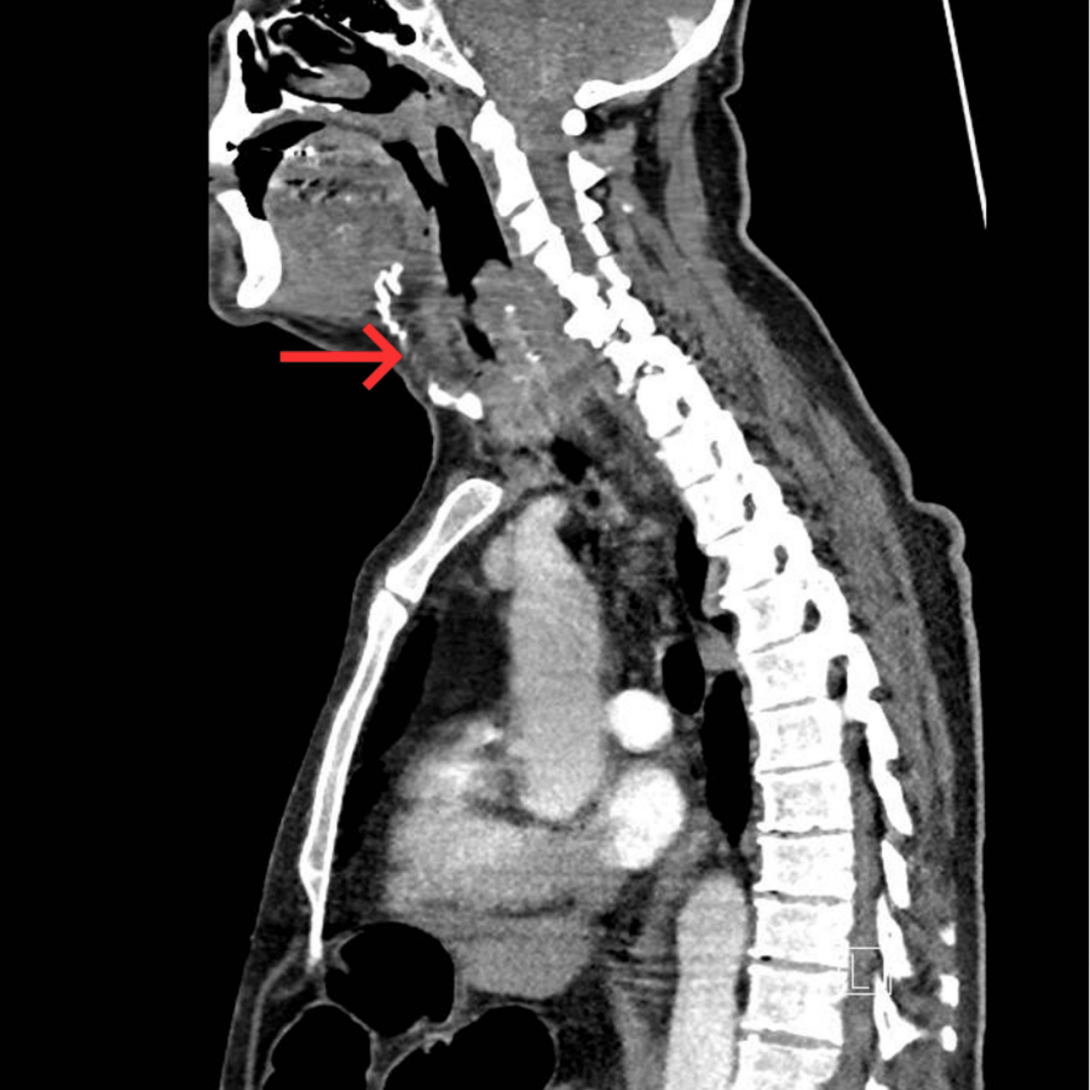
Sagittal CT of the neck demonstrating severe supraglottic and pharyngeal oedema. The red arrow highlights marked soft-tissue swelling with significant narrowing of the upper airway.

Despite the removal of the nasogastric tube due to discomfort, respiratory symptoms progressed. Examination revealed inspiratory stridor without hypoxia. An urgent otolaryngology review was requested, and contrast-enhanced CT of the neck was performed (Figures 1, 2).

On otolaryngology assessment, the patient exhibited increased work of breathing with inspiratory stridor but preserved voice quality. He was unable to mobilise short distances without respiratory distress. Flexible fibreoptic laryngoscopy demonstrated severe supraglottic oedema with bilateral vocal cord immobility. A provisional diagnosis of parapharyngeal infection with paraglottic space involvement was considered.

**Figure 3:**
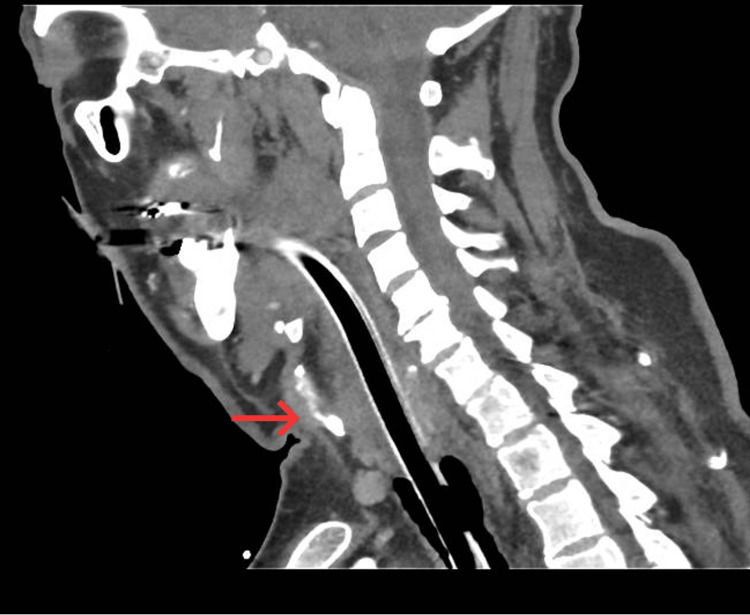
Sagittal CT of the neck demonstrating supraglottic, glottic and subglottic oedema following endotracheal intubation. Red arrows highlight soft-tissue swelling at the supraglottic, glottic and subglottic levels, resulting in airway narrowing.

**Figure 4:**
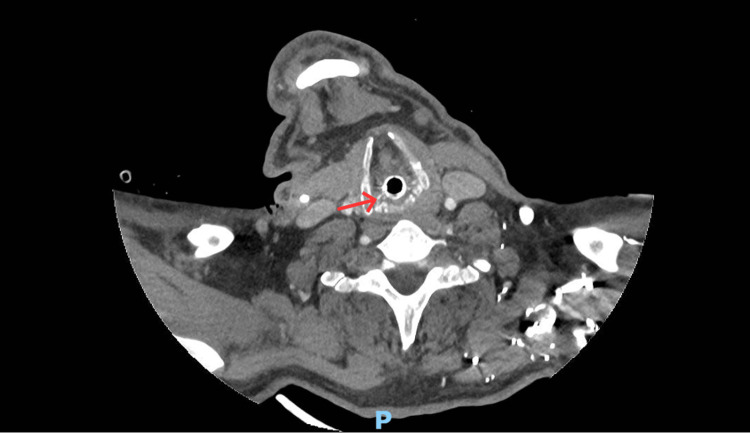
Axial CT of the neck demonstrating vocal cord and paraglottic oedema with the endotracheal tube in situ. The red arrow highlights oedematous thickening of the vocal cord and paraglottic soft tissues, associated with narrowing of the laryngeal airway.

The anaesthesiology team was alerted. The patient was transferred to the intensive care unit and underwent awake fibreoptic intubation. Subsequent contrast-enhanced CT of the neck demonstrated marked supraglottic oedema (Figures 3, 4).

He was taken to the operating theatre for pan-endoscopy to assess the larynx, hypopharynx and oesophagus. Endoscopic examination revealed sloughing over the posterior cricoid lamina with surrounding inflammation, consistent with cricoid chondritis. Significant supraglottic and glottic oedema was noted. Swabs and biopsies were obtained, but were non-diagnostic.

Intravenous ceftriaxone and metronidazole were commenced in consultation with microbiology. The patient remained ventilated in intensive care for five days. With improvement in inflammatory markers and endoscopic findings, repeat pan-endoscopy demonstrated resolution of cricoid inflammation and reduction in supraglottic oedema. He was successfully extubated and monitored with serial endoscopic assessments until vocal cord mobility recovered. He was discharged with a further two-week course of oral antibiotics.

Investigations

Initial abdominal radiography demonstrated delayed progression of oral contrast through the small bowel, consistent with small bowel obstruction. Subsequent imaging showed transit of contrast into the large bowel, correlating with return of stoma output and clinical improvement.

Following the onset of respiratory symptoms, chest radiography demonstrated no focal consolidation or acute pulmonary pathology. Given the presence of inspiratory stridor, flexible nasal endoscopy was performed urgently. This revealed an omega-shaped epiglottis with marked oedema of the arytenoids and false vocal cords, preventing full visualisation of the true vocal cords. There was significant posterior hypopharyngeal wall swelling, resulting in severe supraglottic airway narrowing.

Computed tomography of the neck and thorax demonstrated diffuse circumferential oedema of the hypopharyngeal walls and aryepiglottic folds, with significant luminal narrowing at the supraglottic level. The remainder of the aerodigestive tract was patent, with no evidence of abscess, mass lesion, or cervical lymphadenopathy. The parotid, submandibular, and thyroid glands were unremarkable, and there was no radiological evidence of lower respiratory tract infection.

Laboratory investigations demonstrated a marked inflammatory response, with a peak white cell count of 20.0 ×10⁹/L and C-reactive protein of 189 mg/L, supporting an acute inflammatory process. As shown in Table 1, the peak in inflammatory markers temporally correlated with the development of severe supraglottic oedema, supporting an acute inflammatory pathophysiological process.

**Table 1:** Serial laboratory parameters during clinical progression of nasogastric tube syndrome

Clinical phase	Haemoglobin (g/L)	White cell count (×10⁹/L)	C-reactive protein (mg/L)
Admission	140	14.1	28
Pre-respiratory symptoms	113	13.0	14
Peak airway compromise	116	20.0	189
Post-airway intervention	100	9.7	30
Recovery phase	122	13.5	8
Pre-discharge	111	8.0	6
Follow-up (2 weeks)	123	6.3	11

Differential diagnosis 

The development of inspiratory stridor in a hospitalised surgical patient prompted consideration of several differential diagnoses. Lower respiratory tract infection was initially considered given the elevated inflammatory markers; however, the absence of hypoxia, normal chest radiography and lack of focal respiratory findings made this unlikely as the primary cause of airway compromise.

Acute epiglottitis was considered due to supraglottic involvement; however, the absence of systemic toxicity, preserved epiglottic contour on flexible nasal endoscopy and lack of focal infective changes on imaging argued against this diagnosis. Allergic angioedema was considered but deemed unlikely in the absence of allergen exposure, facial or lip swelling, or rapid response to corticosteroid therapy. Laryngeal malignancy or tumour recurrence was excluded based on the acute onset of symptoms, absence of mass lesions on imaging and rapid resolution with supportive management. In the context of recent nasogastric tube insertion and characteristic endoscopic findings, nasogastric tube syndrome was considered the most consistent diagnosis.

Treatment

Following recognition of upper airway compromise, the nasogastric tube was removed immediately. Initial medical management included nebulised adrenaline (2 mg of 1:1000 solution) and high-dose intravenous dexamethasone (6.6 mg), with close monitoring of respiratory status. Broad-spectrum intravenous antimicrobial therapy was commenced to cover potential secondary infection, including intravenous ceftriaxone and metronidazole. Despite these measures, the patient developed progressive inspiratory stridor and increased work of breathing. He was transferred to the intensive care unit for airway monitoring and escalation of care. Given worsening airway obstruction, awake fibreoptic intubation was performed due to concern regarding loss of airway with standard induction. Significant supraglottic oedema was encountered, and tracheal intubation was achieved with difficulty using a size 5 endotracheal tube. Systemic corticosteroid therapy was continued, with intravenous dexamethasone administered three times daily and subsequently weaned according to clinical and endoscopic improvement. Nutritional support was provided via total parenteral nutrition. Due to persistent airway oedema and anticipated prolonged intubation, a surgical tracheostomy was performed. Antimicrobial therapy was adjusted in consultation with microbiology and discontinued following clinical improvement and exclusion of deep-seated infection.

Outcome and follow-up

Following admission to intensive care, the patient demonstrated gradual clinical improvement with a reduction in supraglottic and hypopharyngeal oedema on serial endoscopic assessment. Repeat flexible nasal endoscopy showed progressive resolution of arytenoid and false vocal cord swelling, with restoration of vocal cord mobility. Cross-sectional imaging confirmed interval improvement and excluded deep neck space infection or mass lesion.

The patient was successfully extubated after clinical and endoscopic improvement, and the tracheostomy was subsequently decannulated. Swallowing assessment initially demonstrated an aspiration risk; however, this improved with speech and language therapy input, allowing gradual reintroduction of oral intake. Corticosteroids were tapered and discontinued without recurrence of symptoms.

At discharge, the patient had normal voice quality, stable respiratory function, and was tolerating an oral diet. Outpatient follow-up with otolaryngology and surgical teams was arranged. At early follow-up, he remained clinically stable with no recurrence of airway symptoms.

## Discussion

Nasogastric tube syndrome is a rare but potentially life-threatening complication of nasogastric intubation that may be overlooked because of its delayed onset and initially subtle clinical features. Patients frequently maintain normal oxygen saturations and the ability to phonate despite significant supraglottic compromise, which may falsely reassure clinicians and delay escalation of care.

In postoperative patients, early symptoms, such as hoarseness, throat discomfort or mild dyspnoea, are often attributed to lower respiratory tract infection, bronchospasm or fluid overload, increasing the risk of delayed diagnosis [1-3].

The proposed pathophysiology involves sustained mechanical pressure exerted by the nasogastric tube on the posterior hypopharyngeal and cricoid mucosa, resulting in local ischaemia, mucosal ulceration and inflammatory oedema affecting the arytenoids, aryepiglottic folds and false vocal cords. Secondary bacterial colonisation may exacerbate inflammation. Anatomical distortion, prolonged tube dwell time, difficult insertion and prior radiotherapy have been suggested as predisposing factors. Importantly, airway oedema may progress even after tube removal [3-5].

Early recognition is essential to prevent catastrophic airway obstruction. Any patient who develops stridor or voice change following nasogastric tube insertion should undergo urgent flexible nasal endoscopy. Cross-sectional imaging is useful to exclude deep neck space infection or malignancy and to delineate the extent of airway involvement [3,4].

Management requires immediate tube removal, early otolaryngology involvement and prompt initiation of systemic corticosteroids with nebulised adrenaline. However, as illustrated in this case and summarised in Table 2, medical therapy alone may be insufficient, and definitive airway protection is frequently required. Increased awareness of nasogastric tube syndrome among surgical, medical and critical care teams is essential to reduce associated morbidity and mortality [1,2,6].

**Table 2:** Reported cases of nasogastric tube syndrome and associated airway outcomes

Author	Patient Context	Immunocompromised	Symptom	Time of Symptom Onset	Airway Intervention	Airway Findings	Outcome
Sanaka et al. [3]	Small bowel obstruction secondary to post-operative adhesions	No	Stridor, Shortness of Breath	4 days	Tracheostomy	B/L Mild arytenoid oedema	Decannulated after 3 weeks
Sano et al. [5]	Large bowel obstruction secondary to sigmoid colon cancer	No	Stridor, throat pain, Hypoxia	6 days	Tracheosotomy	B/L arytenoid oedema	Decannulated after 4 weeks
Apostolakis LW et al. [6]	Toxic megacolon	No	Stridor	2 days	Tracheosotomy	B/L impaired vocal cord abduction bilaterally, with oedema; postcricoid necrotic ulcer was noted, 1.5 cm in width.	No recovery till 1 month

## Conclusions

Nasogastric tube syndrome, although rare, represents a potentially life-threatening complication of an otherwise routine procedure. The onset of respiratory symptoms following nasogastric tube insertion should raise immediate concern and prompt urgent airway assessment. Flexible nasendoscopy plays a crucial role in early recognition and diagnosis. Importantly, removal of the nasogastric tube alone may not halt disease progression once significant airway oedema has developed. Early involvement of a multidisciplinary team and timely airway protection are essential and may be life-saving.
